# Case Reports

**DOI:** 10.4103/0970-0358.41124

**Published:** 2008

**Authors:** Bhagath Kumar Potu

**Affiliations:** Department of Anatomy, Kasturba Medical College, Manipal University, Karnataka - 576 104, India

Dear Sir,

This is regarding Dr. Mukund Thatte's editorial on “A Point Regarding Case Reports”.[1]

I appreciate your respect for case reports. Writing case reports for publication in journals is one of the best ways to get started in the medical profession. Case reports are little mysteries that hold readers’ interest far more effectively than other types of papers like original research and review articles.

An essential feature of a publishable case report is its educational value. It is not true that a report must present a unique finding. Most case reports are published because they support the findings presented in previously published cases or because they are useful reminders of an important point in diagnosis or treatment.

## References

[1] Thatte M (2007). A point regarding case reports. Indian J Plast Surg.

